# Differences in alcohol consumption and drinking patterns in Ghanaians in Europe and Africa: The RODAM Study

**DOI:** 10.1371/journal.pone.0206286

**Published:** 2018-11-02

**Authors:** Juliet Addo, Sarah Cook, Cecilia Galbete, Charles Agyemang, Kerstin Klipstein-Grobusch, Mary Nicolaou, Ina Danquah, Matthias B. Schulze, Rachel Brathwaite, Frank P. Mockenhaupt, Erik Beune, Karlijn Meeks, Ama de-Graft Aikins, Silver Bahendaka, Ellis Owusu-Dabo, Liam Smeeth

**Affiliations:** 1 Department of Non-communicable Disease Epidemiology, Faculty of Epidemiology and Population Health, London School of Hygiene & Tropical Medicine, Keppel Street, London, United Kingdom; 2 Department of Molecular Epidemiology, German Institute of Human Nutrition Potsdam-Rehbruecke, Nuthetal, Germany; 3 Department of Public Health Academic Medical Center, University of Amsterdam, Amsterdam Public Health Research Institute, Amsterdam, The Netherlands; 4 Julius Global Health, Julius Center for Health Sciences and Primary Care, University Medical Center Utrecht, Utrecht University, The Netherlands; 5 Division of Epidemiology & Biostatistics, School of Public Health, Faculty of Health Sciences, University of the Witwatersrand, Johannesburg, South Africa; 6 Institute for Social Medicine, Epidemiology and Health Economics, Charité–Universitaestmedizin Berlin, corporate member of Freie Universitaet Berlin, Humboldt-Universitaet zu Berlin, and Berlin Institute of Health, Berlin, Germany; 7 Institute of Tropical Medicine and International Health, Charité–Universitaestmedizin Berlin, corporate member of Freie Universitaet Berlin, Humboldt-Universitaet zu Berlin, and Berlin Institute of Health, Berlin, Germany; 8 Regional Institute for Population Studies, University of Ghana, Legon-Accra, Ghana; 9 MKPGMS-Uganda Marttyrs University, Kampala, Uganda; 10 Faculty of Science, Kwame Nkrumah University of Science and Technology, Kumasi, Ghana; Monash University, AUSTRALIA

## Abstract

**Background:**

Little is known about alcohol consumption among Africans living in rural and urban Africa compared to African migrants in Europe. We compared the patterns of alcohol consumption in a group of Ghanaians living in different locations in Ghana and in Europe and examined the factors associated with drinking alcohol.

**Methods:**

Data were from a cross-sectional study (RODAM) of Ghanaians aged 25–70 years living in rural and urban Ghana and in Amsterdam, Berlin and London. Information on how often participants consumed at least one standard alcoholic drink in the preceding 12 months, the type of alcoholic beverage and the average serving size was obtained using a food propensity questionnaire. The associations between drinking alcohol and socio-demographic variables, and frequency of attending religious services were investigated using logistic regression models stratified by site and sex. For Ghanaians living in Europe, the number of years since migration and acculturation were also included in the model as covariates.

**Results:**

4280 participants (62.2% women) were included in the analyses. In both men and women, the prevalence of drinking and amount of alcohol consumed per day was highest in Berlin (prevalence of drinking 71.0% and 61.7%) and lowest in urban Ghana (41.4% and 26.8%). After adjustment for age and education in both men and women in Europe, those attending religious services less frequently reported higher levels of drinking alcohol than non-attendants (never attend/no religion compared to attending service at least once a week men OR 4.60 95% CI 2.85, 7.44; women OR 1.80 95% CI 1.12, 2.90) p-trend with frequency <0.001 in men; 0.002 in women); this association was seen also in men in rural Ghana (p-trend = 0.001) and women in urban Ghana (p-trend = 0.02). The prevalence of drinking was positively associated with years since migration in both men and women in Europe ((OR per years increase in time lived in Europe 1.25 (95% CI 1.02,1.53) test for trend p = 0.03 in women; OR 1.29 (95% CI 1.03, 1.62 p = 0.03 in men) but no association was found with self-reported measures of acculturation (ethnic identity, cultural orientation or social networks).

**Conclusion:**

There are marked differences in alcohol consumption between Ghanaians living in Europe and in Ghana suggesting migration has an important influence of drinking patterns and also suggesting the possibility of requiring different strategies in alcohol reduction campaigns among Ghanaians in different locations.

## Introduction

Alcohol consumption is a major public health issue worldwide, accounting for a significant burden of morbidity and mortality annually.[1–3] The harmful effect of alcohol is determined not only by the volume of alcohol consumed, but also by the pattern of drinking and the quality of alcohol consumed.[4–7] The disease burden per litre of alcohol consumed has been shown to be greater in poorer populations and lower-income countries compared to higher income populations and countries.[8]

Several factors have been identified at the individual and societal level to affect the magnitude and patterns of alcohol consumption. These include environmental factors such as economic development, cultural and social factors including religious belief, availability of alcohol and the effectiveness of alcohol policies.[9, 10] A wide variation in alcohol consumption has been reported across world regions and between countries.[3, 11] Globally, the highest levels of alcohol consumption are reported in the European and American regions with intermediate levels reported in the African region.[3] Patterns and types of alcohol consumed may differ not only between countries but within different regions of a country such as urban and rural areas and in migrant groups.[12] Drinking behaviour could also be influenced by drinking-related cultural norms which might differ between migrant and host populations.[13]

Alcoholic beverages have been consumed for generations in many sub Saharan African communities but with considerable variation in the pattern and purpose of consumption.[14] Data on alcohol consumption, drinking patterns and attitudes in sub Saharan countries such as Ghana are generally limited. The total alcohol per capita consumption (in litres of pure alcohol) was estimated to be 7.8 in men and 1.9 in women between 2008–2010 compared to 6.0 overall in the WHO Africa region.[15] This is lower than the 10.9L average consumption reported in the WHO European region. In traditional African societies, alcohol was mainly consumed by male adults, while females and the youth were restrained from drinking, and excess drinking and intoxication attracted negative sanctions.[16] The patterns of alcohol consumption are however reported to be changing with economic development, sociocultural changes and the growth of alcohol industries in recent years in African societies.[14] The type of alcoholic beverage consumed in African societies such as Ghana include locally produced home-brewed beverages with very high alcohol strength and increased health and social consequences such as “akpeteshie”.[17, 18]

Migration and markers of acculturation to the host culture (such as duration of residence, language indicators) in the country of settlement have been shown to influence the consumption of alcohol in some but not all studies.[19] Acculturation is the process of learning that occurs when individuals from a different cultural background are exposed to continuous contact with a new culture.[20] It is a dynamic process by which individuals combine and meld their original and secondary cultures into a third, unique set of cultural values, practices and beliefs.[21] While some previous studies reported alcohol consumption to increase towards higher rates in the host populations particularly in women, others showed a generally lower consumption of alcoholic beverages in migrant groups.[22, 23] Few studies have assessed the effect of migration from rural to urban locations in Africa and in low and middle income countries on alcohol consumption.[12] This study aims to compare the alcohol consumption and drinking patterns in a relatively homogenous population of Ghanaians living in different geographic locations in Europe and in urban and rural Ghana and to examine socio-demographic factors associated with the alcohol consumption in the different locations. For participants in Europe, we additionally assessed the extent to which the duration of residence in Europe and self-reported acculturation influenced these patterns.

## Materials and methods

Data for the current study are based on the RODAM study, a cross-sectional study of a population of Ghanaians living in three European cities (Amsterdam, Berlin and London) and in urban and rural Ghana. The sampling process has been described in detail previously[24]. In brief, between 2012 and 2015, Ghanaians aged between 25 and 70 years residing in the different locations were invited to participate in the study. Different recruitment strategies were used in the various locations to obtain representative samples of Ghanaians. Participants from urban and rural Ghana were randomly drawn from a list of 30 census enumeration areas in the Ashanti region. In Amsterdam, participants were randomly drawn from the Amsterdam Municipal Health register. Ghanaian organisations including churches as well as social and community groups served as the sampling frame in London because of the absence of a register of Ghanaians. In Berlin, a list of Ghanaians was obtained from the registration office of the federal state of Berlin in addition to using a network sampling approach inviting eligible Ghanaians at community gatherings such as religious services and traditional festivals. In each site, data on socio-demographic variables were collected using a standardised questionnaire and data on alcohol consumption were collected using a Ghana-specific food propensity questionnaire.[25] Questionnaires were either self-completed or by face-to-face interviews depending on the preference of the participant. Interviewers were trained to ensure privacy during the interviews. All participants provided informed written consent. Ethical permission for the study was obtained from all sites (Academic Medical Center, University of Amsterdam, London School of Hygiene & Tropical Medicine, Charité- Universitätsmedizin Berlin and Kwame Nkrumah University of Science and Technology) and ethical procedures for the study were reviewed by an independent external ethics advisor.

Alcohol consumption patterns were assessed in response to questions on frequency and serving size of alcoholic beverages (i.e. beer, wine and spirits) in the preceding 12 months. Serving sizes (i.e standard drinks) were given as 1 bottle (0.5L) of beer, 0.25 L of wine (including palm wine) and 8cL of spirits (including spirits traditionally consumed in Ghana (brukutu, pito and akpeteshie). Average serving size options ranged from half a portion to three portions. Answers to these questions were used to categorise participants as drinkers or non-drinkers (no regular beer, wine or spirits consumed in the preceding 12 months), and to investigate frequency of consuming different types of beverage.

Grams of ethanol per day were calculated by multiplying beverage specific frequency and portion size with average beverage strengths for Ghana taken from the West-African food composition tables (beer 3.4g ethanol/100g, wine 8.8/100 g and the liquors or spirits 33.4 g/100 g) and for Europe from the German food composition table (beer 3.5g ethanol/100g, wine 8.6/100 g and the liquors or spirits 18 g/100 g). Since the maximum portion size that participants could report was 3 portions, this variable was considered as a potential underestimate of participants’ intake if they were heavier drinkers. To understand the relative possible bias due to under-reporting, participants were additionally categorised by whether they reported three or more portions for any beverage (beer, wine or spirits) or not.

Socio-demographic variables assessed included age, education, and marital status. The level of education based on the highest qualification gained either in Ghana or elsewhere was classified into three groups as elementary (never been to school or primary/elementary schooling only), secondary (lower or intermediate vocational schooling or lower secondary schooling) and higher education (higher secondary education/higher vocational schooling, post-secondary education or university education). Marital status was categorised as married, cohabiting, never married, divorced/separated and widowed.

Other variables of interest were frequency of attending religious services, psycho-social distress, and smoking status. Smoking status was categorised as never smoker, ex-smoker and current smoker. Participants were asked if they were currently practicing a specific religion and the number of times they had attended a religious service in the preceding 6 months including attendance to church, mosque or synagogue. The level of psychosocial stress was assessed from a combined score measured by a psychological stress scale created by the INTERHEART study[26] which distinguished between participants’ level of stress at work and at home (never experienced stress/ experienced some periods of stress at work or at home or experienced several periods of stress/permanent stress at work or at home).

Information on the number of years participants had lived in Europe was obtained for those who had migrated based on the response to the following question: “What is your country of birth?” and “What year did you come to live in the UK, Germany or the Netherlands?” Level of acculturation for participants living in Europe was assessed using Berry’s bi-dimensional perspective on three domains–two psychological (ethnic identity, cultural orientation) and one behavioural (social networks)[27]. For each domain participants were categorised with regard to integration (adaptation to host culture without loss of ethnic identity), assimilation (cultural adaptation to the host culture accompanied by loss of original culture), separation (rejection of the host culture and orientation to the original culture) and marginalisation (rejection of both host culture and culture of origin). Ethnic identity was assessed from two questions on a Likert scale “I feel Dutch/German/English” and” I feel Ghanaian” with a score ≥3. Cultural orientation was assessed by the Psychological Acculturation Scale (PAS) developed by Stevens et al.[28] Social networks were assessed by 4 items–a) the extent to which participants had Dutch/German/English friends and b) the extent to which they have friends of their country of origin and the extent to which they spent their free time c) with Dutch/German/English people and d) with people of their own ethnicity. Given very small numbers of participants in the assimilation and marginalized categories a binary categorization of more acculturation (integrated+assimilated) and less acculturation (separated+marginalized) was used for each variable.

Since the focus of this study was migration, a small number of participants (n = 75) from the European sites who were not first generation migrants or who did not report country of birth were excluded from the analyses.

### Statistical analysis

The prevalence of different aspects of drinking pattern (prevalence of drinking alcohol, frequency of consuming different beverages, and volume of ethanol consumed) were calculated separately by sex and geographic location.

Given that overall prevalence of drinking was low particularly among women, analysis of associations with drinking were investigated only for the outcome drinking alcohol. Factors associated with drinking were investigated by fitting logistic regression models with drinking status as the outcome and age, education, marital status, psychosocial distress, smoking and frequency of attending religious services as exposures. Models were adjusted for 1) age and 2) age and education. Due to small numbers and to limit the number of comparisons, the three sites in Europe were combined together for the regression analysis and site adjusted for as a confounder for the models for the European sites. As a final stage all exposure variables were included in the model to understand which variables were independently associated with drinking however given missing data on co-variates and difficulty of untangling the direction of effects in cross-sectional analyses these models are reported separately as supplementary material. All models were stratified by sex and study site based on a priori interest in whether associations were the same in each location and by gender. For Europe, the number of years since migration and acculturation were also included in the model as covariates. To investigate the validity of grouping the three European sites together interactions between European city and each exposure in the models adjusted for age and education were tested for using likelihood ratio tests. For the logistic regression models a complete case analysis approach was used. Analyses were restricted for all models to those with data on age and education available (n = 124 participants with missing data on education were excluded from the logistic regression analyses).

## Results

For the current analysis 4280 RODAM study participants born in Ghana, aged 25–70 with information on alcohol consumption were included. Of these, 1949 reported consuming any alcoholic beverage in the past 12 month (45.5%). The proportion of participants completing the Ghana-specific food propensity questionnaire were 99.2% Rural Ghana, 98.5% Urban Ghana, 42.6% London; 97.4% Berlin and 63.4% Amsterdam. There were no differences between those completing the food frequency questionnaire in Berlin, urban Ghana and rural Ghana but there was evidence for difference in marital status, frequency of attending religious services and years since migration for the participants from Amsterdam and for age and frequency of attending religious services for participants from London (S1 Table).

The distribution of drinking variables in men and women are shown in Table 1. The majority of the study participants were women (62.2%). The distribution of socio-demographic factors by sex and geographic location are shown in S1 and S2 Tables.

**Table 1:** Distribution of alcohol drinking behaviours among men and women by study site.

		Amsterdam		Berlin		London		Urban Ghana		Rural Ghana	
	**Males**	**(n = 359)**		**(n = 290)**		**(n = 170)**		**(n = 408)**		**(n = 392)**	
Drinking Status (drank regular beer, wine or spirits in the past 12 months)	Non-drinker	116	(32.3)	84	(29.0)	85	(50.0)	237	(58.1)	164	(41.8)
	Drinker	243	(67.7)	206	(71.0)	85	(50.0)	171	(41.9)	228	(58.2)
Frequency of drinking beer among drinkers	Never drinks beer	58	(23.9)	28	(13.6)	35	(41.2)	31	(18.1)	76	(33.3)
	Monthly	97	(39.9)	80	(38.8)	35	(41.2)	113	(66.1)	127	(55.7)
	Weekly	833	(34.2)	90	(43.7)	15	(17.7)	25	(14.6)	23	(10.1)
	Daily	5	(2.1)	8	(3.9)	0	(0)	2	(1.2)	2	(0.9)
Frequency of drinking wine among drinkers	Never drinks wine	27	(11.1)	28	(13.6)	4	(4.7)	104	(60.8)	107	(46.9)
	Monthly	136	(56.0)	130	(63.1)	71	(83.5)	62	(36.3)	107	(46.9)
	Weekly	78	(32.1)	47	(22.8)	10	(11.8)	5	(2.9)	12	(5.3)
	Daily	2	(0.8)	1	(0.5)	0	(0)	0	(0)	2	(0.9)
Frequency of drinking liquor or spirits among drinkers	Never drinks spirits	118	(48.6)	67	(32.5)	48	(56.5)	74	(43.3)	69	(30.3)
	Monthly	91	(37.5)	71	(34.4)	28	(32.9)	73	(42.7)	86	(37.7)
	Weekly	34	(14.0)	64	(31.1)	9	(10.6)	21	(12.3)	51	(22.4)
	Daily	0	(0)	4	(1.9)	0	(0)	3	(1.8)	22	(9.7)
Median average consumption per day in grams ethanol among drinkers (IQR)		6.2	(2.5–15.9)	9.5	(2.9–36.2)	1.7	(1.0–6.7)	1.6	(0.8–4.1)	2.7	(1.2–8.2)
Number of participants reporting 3 or more portions of beer/ / wine or spirits per occasion among drinkers		27	(11.1)	50	(24.3)	5	(5.9)	17	(9.9)	46	(20.2)
	**Females**	**(n = 551)**		**(n = 240)**		**(n = 255)**		**(n = 1013)**		**(n = 602)**	
Drinking Status (drank regular beer, wine or spirits in the past 12 months)	Non-drinker	252	(45.7)	92	(38.3)	146	(57.3)	742	(73.3)	413	(68.6)
	Drinker	299	(54.3)	148	(61.7)	109	(42.8)	271	(26.8)	189	(31.4)
Frequency of drinking beer among drinkers	Never drinks beer	134	(44.8)	52	(35.1)	57	(52.3)	64	(23.6)	110	(58.2)
	Monthly	120	40.1)	75	(50.7)	46	(42.2)	187	(69.0)	72	(38.1)
	Weekly	44	(14.7)	19	(12.8)	6	(5.5)	20	(7.4)	7	(3.7)
	Daily	1	(0.3)	2	(1.4)	0	(0)	0	(0)	0	(0)
Frequency of drinking wine among drinkers	Never drinks wine	16	(5.4)	14	(9.5)	11	(10.1)	197	(72.7)	92	(48.7)
	Monthly	210	(70.2)	102	(68.9)	83	(76.2)	66	(24.4)	90	(47.6)
	Weekly	73	(24.4)	32	(21.6)	15	(13.8)	8	(3.0)	7	(3.7)
	Daily	0	(0)	0	(0)	0	(0)	0	(0)	0	(0)
Frequency of drinking liquor or spirits among drinkers	Never drinks spirits	252	(84.3)	92	(62.2)	86	(78.9)	170	(62.7)	80	(42.3)
	Monthly	36	(12.0)	36	(24.3)	19	(17.4)	75	(27.7)	92	(48.7)
	Weekly	11	(3.7)	20	(13.5)	4	(3.7)	26	(9.6)	14	(7.4)
	Daily	0	(0)	0	(0)	0	(0)	0	(0)	3	(1.6)
Median average consumption per day in grams ethanol among drinkers (IQR)		2.3	(1.3–6.2)	2.0	(1.3–8.6)	1.4	(0.7–3.5)	1.2	(0.6–2.0)	1.2	(0.6–2.0)
Number of participants reporting 3 or more portions of beer/ wine or spirits per occasion among drinkers		10	(3.3)	10	(6.8)	4	(3.7)	10	(3.7)	17	(9.0)

### Patterns of alcohol consumption by geographic location in men and women

The distribution of drinking behaviours by geographic location and sex are shown in Table 1. In all geographic locations the prevalence of drinking and the median amount of ethanol consumed per day among drinkers were higher in men than in women.

Among men the prevalence of drinking alcohol and the median amount consumed per day were highest in Berlin, followed by Amsterdam while the lowest levels were in Urban Ghana. London was similar to Urban Ghana while rural Ghana was in between Urban Ghana and the European cities (with the exception of London). In most sites, the most commonly consumed beverage was beer with the exception of London where drinkers were more likely to drink wine. Wine consumption was more common in all three European cities compared to Ghana. In all sites daily drinking of any beverage was rare but in rural Ghana daily consumptions of spirits (9.7%) was higher as compared to all other sites (<2%).

Geographic patterns of drinking behaviours were similar for women with a higher prevalence of drinking and median amount of ethanol / day in Berlin and Amsterdam compared to the other sites. The lowest prevalence of drinking was observed for Urban Ghana (26%). In contrast to men, the drinking profile of women in Rural Ghana was similar to Urban Ghana, whilst women in London consumed more alcohol than women in Ghana but less than women in Amsterdam and Berlin. As with men, amongst women drinking wine was less common in Ghana than in Europe, whilst drinking spirits was more common in rural Ghana than in the other sites. Daily consumption of any beverage was very rare in all sites.

### Factors associated with alcohol consumption by sex and geographic location

Table 2 shows associations between socio-demographic factors and drinking status in men. After adjustment for age and education, drinkers were more likely to attend religious services less frequently or not at all (OR per decreasing level of frequency 1.78 (95% 1.52, 2.09) p-trend <0.001)) compared to non-drinkers among Ghanaians living in Europe. The odds of drinking were lowest among men who attended religious services once a week or more, compared to those who attended less frequently (OR 4.60 no religion/never attend compared to attending once a week 95% CI 2.85, 7.44). The increased odds of drinking among men with no religion/never attend religious services was also observed in rural but not urban Ghana (OR 2.17 95% CI 1.35, 3.47). Men in rural Ghana had higher odds of drinking if they were divorced or separated compared to those who were married (OR 3.62 95% CI 1.42, 9.19). Smoking and drinking were associated in all sites but the pattern of association was different. Among male Ghanaians living in Europe and in rural Ghana there was strong evidence for an association between smoking and drinking (p<0.001). Among male Ghanaians living in Europe the odds of drinking were higher in current smokers compared to never smokers (OR 7.85 95% CI 3.10, 19.89) and among men living in rural Ghana odds of drinking were 5.73 (95% CI 1.63, 20.12) times higher in current smokers and also raised to a lesser extent (OR 2.07 95% CI 1.07, 3.98) in ex-smokers compared to never smokers. Among men living in Urban Ghana the association between smoking and drinking was weaker with only some evidence for higher odds of drinking in ex-smokers compared to never smokers (OR 1.97 95%C (1.10, 3.52) and no evidence for higher odds of drinking in current smokers (OR 2.02 95% CI 0.64,6.33). There was no evidence of an association between drinking and psycho-social stress in any site.

**Table 2:** Association between drinking alcohol with socio-demographic and behavioural factors and measures of acculturation in men.

		Europe (N = 813)				Urban Ghana (n = 387)				Rural Ghana (n = 369)			
		Adjusted for age and site		Adjusted for age, education and site		Adjusted for age		Adjusted for age and education		Adjusted for age		Adjusted for age and education	
		OR	(95% CI)	OR	(95% CI)	OR	(95% CI)	OR	(95% CI)	OR	(95% CI)	OR	(95% CI)
Age	25–34	1.21	(0.71, 2.05)	1.20	(0.71, 2.04)	1.07	(0.57,2.01)	1.02	(0.54,1.93)	1.13	(0.61, 2.08)	1.17	(0.63,2.18)
	35–44	1.00	(ref)	1.00	(ref)	1.00	(ref)	1.00	(ref)	1.00	(ref)	1.00	(ref)
	45–54	1.13	(0.76,1.67)	1.14	(0.77, 1.68)	0.71	(0.40, 1.26)	0.69	(0.39, 1.24)	1.07	(0.59,1.92)	1.05	(0.58,1.90)
	55–70	1.57	(1.05, 2.37)	1.58	(1.05, 2.38)	0.85	(0.49,1.49)	0.85	(0.48,1.49)	1.31	(0.75, 2.31)	1.31	(0.75, 2.32)
	Test for linear trend	P = 0.09		P = 0.08		P = 0.32		P = 0.37		P = 0.52		P = 0.60	
Education	Never been to school/elementary school	1.18	(0.75,1.86)	1.18	(0.75,1.86)	1.55	(0.94, 2.58)	1.55	(0.94, 2.58)	0.91	(0.58,1.40)	0.91	(0.58,1.40)
	Lower vocational school/secondary	1.00	(ref)	1.00	(ref)	1.00		1.00		1.00		1.00	
	Higher level/university	1.15	(0.66, 2.02)	1.15	(0.66, 2.02)	1.45	(0.66,3.20)	1.45	(0.66,3.20)	0.59	(0.24,1.45)	0.59	(0.24,1.45)
	Test for linear trend	P = 0.67		P = 0.67				P = 0.18		P = 0.33		P = 0.33	
Marital status (missing = 16)	Married	1.00	(ref)	1.00	(ref)	1.00	(ref)	1.00	(ref)	1.00	(ref)	1.00	(ref)
	Cohabiting	1.48	(0.92, 2.39)	1.48	(0.92, 2.39)	1.39	(0.60, 3.23)	1.49	(0.64, 3.51)	1.27	(0.72, 2.23)	1.25	(0.71, 2.22)
	Never Married	1.27	(0.83, 1.97)	1.27	(0.82, 1.96)	0.98	(0.47,2.03)	0.95	(0.46, 1.99)	0.91	(0.38, 2.17)	0.96	(0.40,2.32)
	Divorced/Separated	0.99	(0.63, 1.56)	1.00	(0.64, 1.56)	1.11	(0.45,2.73)	1.14	(0.46, 2.83)	3.67	(1.45,9.30)	3.62	(1.42, 9.19)
	Widowed	3.48	(0.39, 30.88)	3.48	(0.39, 31.0)	0.79	(0.07, 8.85)	0.72	(0.06, 8.13)	2.39	(0.24,23.43)	2.33	(0.24, 22.92)
	Test for heterogeneity	P = 0.30		P = 0.31		P = 0.95		P = 0.90		P = 0.04		P = 0.05	
Frequency of attending religious service (missing = 34)	Once a week	1.00	(ref)	1.00	(ref)	1.00	(ref)	1.00	(ref)	1.00	(ref)	1.00	(ref)
	At least once a month but not every week	3.86	(2.23, 6.69)	3.88	(2.34, 6.74)	1.14	(0.30, 4.39)	1.16	(0.30, 4.49)	2.37	(0.80,6.99)	2.33	(0.79, 6.91)
	Less than once a month	4.36	(1.99,9.56)	4.39	(2.00, 9.63)	0.70	(0.12,3.94)	0.69	(0.12, 3.91)	1.77	(0.32, 9.90)	1.88	(0.33, 10.74)
	Never /No current religion	4.61	(2.85, 7.45)	4.60	(2.85, 7.44)	1.40	(0.90,2.18)	1.48	(0.94, 2.33)	2.14	(1.34, 3.42)	2.17	(1.35, 3.47)
	Test for linear trend	P<0.001		P<0.001		P = 0.16		P = 0.10		P = 0.001		P = 0.001	
Smoking status (missing = 1)	Never smoker	1.00	(ref)	1.00	(ref)	1.00	(ref)	1.00	(ref)	1.00	(ref)	1.00	(ref)
	Current smoker	7.61	(3.01, 19.23)	7.85	(3.10,19.89)	1.83	(0.60, 5.64)	2.02	(0.64,6.33)	5.67	(1.63, 19.77)	5.73	(1.63, 20.12)
	Ex-smoker	1.23	(0.77, 1.97)	1.23	(0.76, 1.97)	1.91	(1.08, 3.39)	1.97	(1.10,3.52)	2.08	(1.09,3.97)	2.07	(1.07, 3.98)
	Test for heterogeneity	P<0.001		P<0.001		P = 0.06		P = 0.04		P = 0.001		P = 0.001	
Psycho-social stress (missing = 19)	Never experience stress	1.00	(ref)	1.00	(ref)	1.00	(ref)	1.00	(ref)	1.00	(ref)	1.00	(ref)
	Some periods of stress and home or work	1.35	(0.98, 1.86)	1.35	(0.98, 1.87)	0.81	(0.51, 1.26)	0.85	(0.54, 1.33)	1.26	(0.77, 2.08)	1.26	(0.77, 2.07)
	Several periods or stress at home or work/permanent stress at home or work	1.17	(0.73, 1.87)	1.19	(0.74, 1.91)	1.24	(0.68, 2.27)	1.33	(0.72,2.46)	1.62	(078,3.35)	1.61	(0.77, 3.35)
	Test for linear trend	P = 0.19		P = 0.17		P = 0.77		P = 0.59		P = 0.18		P = 0.18	
Years since Migration (missing = 57)	1–5 years	0.61	(0.39, 0.98)	0.62	(0.39, 0.98)								
	5–9 years	0.66	(0.39, 1.12)	0.66	(0.39, 1.13)								
	10 or more years	1.00	(ref)	1.00	(ref)								
	Test for linear trend	P = 0.02		P = 0.03									
Acculturation (ethnic identity)	More acculturated	1.00	(ref)	1.00	(ref)								
	Less acculturated	0.73	(0.54, 0.99)	0.73	(0.54, 0.99)								
Acculturation (cultural orientation)	More acculturated	1.00	(ref)	1.00	(ref)								
	Less acculturated	0.90	(0.64,1.27)	0.91	(0.64, 1.29)								
Acculturation (social networks)	More acculturated	1.00	(ref)	1.00	(ref)								
	Less acculturated	1.04	(0.74, 1.45)	1.04	(0.74, 1.45)								

The findings for women are shown in Table 3. There was some evidence that drinking was more common in older women (OR per increase in age group 1.34 (95% CI 1.16, 1.55) test for trend p = 0.001) compared to younger women in Europe. Similar to men, after adjusting for age and education, women in Europe were less likely to be drinkers if they attended a religious service frequently (OR per decreasing level of frequency 1.27 (95% 1.09, 1.48) p-trend = 0.002) although the effect size was smaller than in men. There was some evidence that odds of drinking were also higher in women in urban Ghana who never attend a religious service or have no current religion (OR 1.46 95%CI 1.05, 2.01). Associations in urban and rural Ghana were different to women living in Europe. After adjustment for age there was good evidence of an association between education and drinking status in women in urban Ghana with higher levels of drinking among the less educated (OR for never went to school /elementary compared to secondary education 1.42 95% CI 1.06, 1.90) but conversely with a test for trend (p = 0.007) indicating increased odds of drinking as education category increased (1.42 increase in odds of drinking per increase in educational group 95% CI 1.10,1.84). For marital status there were higher levels of drinking in those who were cohabiting compared to those who were married (OR 2.00 95% CI 1.22, 3.26). Among women in rural Ghana there was evidence for a trend in age with higher odds of drinking in younger women (OR for 1 unit increase in age group 0.83 (95% CI 0.71, 0.98) test for trend p = 0.03) and for an association with marital status overall (p = 0.05) although the confidence intervals for all odds ratios crossed 1. As with men there was strong evidence for an association with smoking among Ghanaian women living in Europe (P<0.001) with higher odds of drinking in current (OR 10.57 95% CI 1.37, 81.55) and ex-smokers (2.35 95%CI 1.22, 4.51) compared to never smokers. The number of current smokers among women living in rural (0) and urban Ghana (1) was too low to assess association between smoking and drinking but there was evidence in both sites for increased odds of drinking in ex-smokers compared to drinkers. There was no evidence for an association between psycho-social stress and drinking in rural and urban Ghana but there was some evidence for increased odds of drinking among those with some periods of stress compared to no stress among Ghanian women living in Europe (OR 1.38 95% CI 1.04, 1.82).

**Table 3:** Association between drinking alcohol with socio-demographic and behavioural factors and measures of acculturation in women.

		Europe(n = 1033)				Urban Ghana(n = 994)				Rural Ghana (n = 560)			
		Adjusted for age and site		Adjusted for age, education and site		Adjusted for age		Adjusted for age and education		Adjusted for age		Adjusted for age and education	
Age	25–34	0.93	(0.58, 1.50)	0.92	(0.57, 1.49)	0.65	(0.42, 1.00)	0.60	(039,0.93)	0.92	(0.54,1.54)	0.96	(0.56,1.63)
	35–44	1.00	(ref)	1.00	(ref)	1.00	(ref)	1.00	(ref)	1.00	(ref)	1.00	(ref)
	45–54	1.50	(1.11, 2.03)	1.52	(1.12, 2.06)	0.92	(0.64,1.32)	0.93	(0.65,1.34)	0.70	(0.43,1.14)	0.70	(0.43, 1.15)
	55–70	1.92	(1.35, 2.75)	1.94	(1.35, 2.77)	0.68	(0.46,1.01)	0.71	(0.48,1.05)	0.59	(0.36, 0.98)	0.60	(0.36,0.99)
	Test for linear trend	P = 0.001		P<0.001		P = 0.84			P = 0.79	P = 0.04		P = 0.03	
Education	Never been to school/elementary school	1.11	(0.83, 1.50)	1.11	(0.83, 1.50)	1.42	(1.06, 1.90)	1.42	(1.06, 1.90)	0.90	(0.61, 1.33)	0.90	(0.61, 1.33)
	Lower vocational school/secondary	1.00	(ref)	1.00	(ref)	1.00	(ref)	1.00	(ref)	1.00	(ref)	1.00	(ref)
	Higher level/university	1.11	(0.66, 1.86)	1.11	(0.66, 1.86)	2.02	(0.90,4.55)	2.02	(0.90,4.55)	0.41	(0.09, 1.93)	0.41	(0.09, 1.93)
	Test for linear trend	P = 0.54		P = 0.54		P = 0.007		P = 0.007		P = 0.33		P = 0.33	
Marital status (missing = 39)	Married	1.00	(ref)	1.00	(ref)	1.00	(ref)	1.00	(ref)	1.00	(ref)	1.00	(ref0
	Cohabiting	1.20	(0.76, 1.90)	1.20	(0.76, 1.90)	1.82	(1.12,2.94)	2.00	(1.22,3.26)	1.44	(0.88,2.37)	1.43	(0.86,2.35)
	Never Married	1.27	(0.87, 1.84)	1.27	(0.87, 1.84)	1.67	(0.91,3.06)	1.61	(0.87, 2.97)	0.31	(0.09, 1.11)	0.31	(0.08,1.12)
	Divorced/Separated	1.29	(0.91, 1.81)	1.29	(0.92, 1.81)	1.36	(0.93, 2.01)	1.37	(0.93,2.03)	1.24	(0.73, 2.12)	1.23	(0.72,2.10)
	Widowed	1.28	(0.56, 2.92)	1.29	(0.57, 2.93)	1.18	(0.73,1.90)	1.23	(0.76, 1.99)	1.78	(1.01, 3.13)	1.76	(1.00,3.11)
	Test for heterogeneity	P = 0.63		P = 0.62		P = 0.08		P = 0.05		P = 0.04		P = 0.05	
Frequency of attending religious service (missing = 68)	Once a week	1.00	(ref)	1.00	(ref)	1.00	(ref)	1.00	(ref)	1.00	(ref)	1.00	(ref)
	At least once a month but not every week	1.43	(0.91, 2.26)	1.43	(0.91, 2.26)	1.97	(0.86,4.48)	2.09	(0.91, 4.79)	1.75	(0.78,3.91)	1.71	(0.76, 3.83)
	Less than once a month	3.16	(1.13, 8.82)	3.11	(1.11, 8.70)	1.06	(0.20,5.57)	0.93	(0.18, 4.91)	0.58	(0.12,2.84)	0.57	(0.12, 2,80)
	Never /No current religion	1.79	(1.11, 2.88)	1.80	(1.12, 2.90)	1.42	(1.03,1.96)	1.46	(1.05, 2.01)	0.87	(0.58,1.30)	0.87	(0.58, 1.30)
	Test for linear trend	P = 0.002		P = 0.002		P = 0.03		P = 0.02		P = 0.47		P = 0.47	
Smoking status (missing = 8)	Never smoker	1.00	(ref)	1.00	(ref)	1.00	(ref)	1.00	(ref)	1.00	(ref)	1.00	(ref)
	Current smoker	10.62	(1.38, 81.75)	10.57	(1.37, 81.55)	Perfect prediction		Perfect prediction		-		-	
	Ex-smoker	2.35	(1.22, 4.51)	2.34	(1.22, 4.50)	2.33	(0.95, 5.70)	2.49	(1.01,6.15)	9.45	(1.04, 86.03)	9.32	(1.02,85.02)
	Test for heterogeneity	P = 0.0003		P = 0.0003		P = 0.07		P = 0.05		P = 0.02		P = 0.02	
Psycho-social stress (missing = 35)	Never experience stress	1.00	(ref)	1.00	(ref)	1.00	(ref)	1.00	(ref)	1.00	(ref)	1.00	(ref)
	Some periods of stress and home or work	1.37	(1.04, 1.82)	1.38	(1.04, 1.82)	1.09	(0.79, 1.50)	1.17	(0.85,1.63)	0.89	(0.57,1.39)	0.86	(0.55, 1.35)
	Several periods of stress at home or work/ Permanent stress at home or work	1.12	(0.78, 1.60)	1.12	(0.78,1.60)	1.25	(0.80,1.50)	1.41	(0.89, 2.24)	0.93	(0.54, 1.59)	0.88	(0.51, 1.52)
	Test for trend	P = 0.21		P = 0.21		P = 0.34		P = 0.14		P = 0.77		P = 0.64	
Years since Migration (missing = 70)	0–4 years	0.67	(0.44, 1.03)	0.68	(0.44,1.04)								
	5–9 years	0.71	(0.49, 1.02)	0.71	(0.49, 1.03)								
	10 or more years	1.00	(ref)	1.00	(ref)								
	Test for linear trend	P = 0.02		P = 0.03									
Acculturation (ethnic identity)	More acculturated	1.00	(ref)	1.00	(ref)								
	Less acculturated	0.92	(0.71, 1.20)	0.92	(0.71, 1.20)								
Acculturation (cultural orientation)	More acculturated	1.00	(ref)	1.00	(ref)								
	Less acculturated	0.84	(0.63, 1.11)	0.84	(0.63, 1.12)								
Acculturation (social networks)	More acculturated	1.00	(ref)	1.00	(ref)								
	Less acculturated	1.13	(0.85, 1.50)	1.13	(0.85, 1.50)								

For both male and female Ghanaians living in Europe years since migration were associated with a higher odds of drinking in age- and education adjusted analysis (OR per years increase in time lived in Europe 1.25 (95% CI 1.02,1.53) test for trend p = 0.03 in women; OR 1.29 (95% CI 1.03, 1.62 p = 0.03 in men). There was no evidence for an association between drinking and any of the three acculturation variables in either sex.

The pattern of results remained similar in regression models with mutual adjustment for all co-variables although there was attenuation of the odds ratios (S4 Table). After mutual adjustment for other variables the association with psycho-social stress and drinking among women in Europe was no longer found.

There was no evidence for interaction between drinking status and any co-variates with the exception of years since migration. There was evidence for an interaction (p = 0.02) for both sexes combined between drinking status and years since migration found for the London site (OR for linear relationship 1.79 95% CI 1.24, 2.58) but not for the Amsterdam (OR for linear relationship 1.10 95% CI 0.88, 1.37) or Berlin sites ((OR for linear relationship 1.14 95% CI 0.88, 1.48). When stratified by sex the evidence for interaction by site and years of migration was weaker (men p = 0.07; women p = 0.06).

## Discussion

The RODAM study presented an opportunity to investigate patterns of alcohol use among Ghanaians living in different geographic locations. We found drinking patterns among Ghanaian adults to differ both by geographic location and by gender. Ghanaians living in Amsterdam and Berlin were more likely to consume alcohol and to have higher intakes compared to those living in Ghana. Within Ghana, alcohol consumption was more common in rural than urban areas. In both urban and rural Ghana, the prevalence of drinking alcohol was lower compared to Ghanaians in Europe, particularly in women. This supports the findings of a previous study comparing NCD risk factors in urban, rural and migrant populations showed a significantly lower alcohol consumption in urban compared to rural groups in China and Ghana whereas urban participants in South Africa were significantly more likely to consume alcohol regularly compared to rural participants.[12] In this study we also found that the types of alcoholic beverage consumed were different: in both men and women beer was the most frequently consumed beverage in urban Ghana while spirits were the most frequently consumed beverage type in rural Ghana. Wine consumption was much more frequent in the three European cities, especially in women. Similar to reports from previous studies, the prevalence of drinking and the volume of alcohol consumed was lower in Ghanaian women in all locations compared to men.[3, 29, 30] The gender ratios in the prevalence of drinking however narrowed among the Ghanaian migrant populations in Europe. The results on frequency of consuming different types of beverage may possibly reflect differences in drinking cultures and availability of particular beverages in different locations as well as the affordability of these beverages.[31]

In line with findings from other migrant populations, we found evidence for a relationship between time since migration and drinking status. [32] Studies involving migrant populations have shown a relationship between acculturation and alcohol use.[13, 19] However, none of the measures of self-reported acculturation showed any association with drinking in our study. While our findings suggest that patterns of alcohol consumption among Ghanaians may be influenced by the patterns of alcohol consumption of the host population, perceptions of cultural identity or social networks (having more friends from the adopted culture) did not seem to explain this. The reasons for these findings cannot be established from the data available and further research is needed to understand this in greater detail.

In traditional African societies, alcohol was mainly consumed by male adults, while females and the youth were restrained from drinking, and excess drinking and intoxication attracted negative sanctions.[16] The prevalence of alcohol consumption in African women has been shown to vary from 1% in Malawi to 30% in Burkina Faso with 81% of African women reporting lifetime abstinence.[33] The prevalence of current drinking within Ghana found in this study is similar to levels reported for the whole of the WHO African region in 2012 (40.2% in men and 19.6% in women)[34]. Data from the WHO European Status report on alcohol and health show that in 2010 the prevalence of abstaining from alcohol in the preceding 12 months was 19.7% in Germany, 11.8% in The Netherlands and 16% in the United Kingdom[35]. Therefore although drinking alcohol was more common in Ghanaians living in Amsterdam and Berlin than in Ghana, it was still lower than the national averages of their host European populations possibly reflecting the maintenance of cultural standards to an extent, even after migration[27].

Various explanations have been suggested for variations in alcohol drinking patterns. These have been reported to differ by age, gender and by country.[36] In this study of Ghanaians in different locations, associations with drinking status differed by geographic location and gender. The most striking result we found was that in both men and women living in the European cities, there was strong evidence that those attending religious services at least once a week were less likely to drink alcohol. This is similar to findings from previous studies reporting a protection from alcohol drinking in people who have a religious belief compared to those who don’t.[37] Interestingly, this strong association between attending religious services and drinking status in our study was not found as strongly or consistently within Ghana. The reason for this is unclear but we hypothesised this could be because drinking is less culturally acceptable in Ghana overall, therefore the influence of personal religious beliefs is not as strong a factor as in Europe where drinking is more common and socially acceptable. It is also important to note that in Ghana the number of participants with a religion who attended a service less frequently than once a week was very small. Religion and upbringing have been reported to influence the choice not to drink in countries with high rates of abstaining.[36]

In women in urban Ghana, associations were found between drinking status and education and relationship status (higher prevalence of drinking in those who were cohabiting). In men, drinking was more common among those who were divorced or separated in rural Ghana but this association was not seen in urban Ghana. These heterogeneous associations are difficult to explain based only on this data but show that alcohol consumption patterns and factors driving them vary within Ghana as well as among Ghanaians who have migrated to Europe. Different patterns between alcohol consumption and socioeconomic status have previously been reported between countries.[38] There are suggestions that while there are more drinkers in the higher socioeconomic groups, people of lower socioeconomic status may be more at risk of severe alcohol-related health outcomes.[3, 39]

There are some limitations which should be considered in the interpretation of these findings. Firstly, the recruitment methods between sites were different which may have introduced some selection bias to the study, in particular with respect to the findings from London where participants were recruited from Ghanaian organisations including churches. The questionnaires were either self- completed or completed by face-to-face interviews with the possibility of introducing social desirability bias. This possibility was minimised by using trained interviewers who were not local to the study area and ensuring privacy during the interviews. In addition, not all participants in the study completed the questions in the Ghana-specific food propensity questionnaire (this affected particularly London and Amsterdam) and therefore did not have data on alcohol consumption. Selection bias for the findings from Europe is possible due to this non-response if it was related to drinking status. This seems plausible as there were differences in the characteristics of participants from London and Amsterdam who completed the food-frequency questionnaire compared to those who did not. This could have biased the findings for the European sites but not for Ghana where the amount of missing data on alcohol consumption was extremely small.

Measurement of alcohol consumption in this study was limited to questions on frequency and usual volume of ethanol consumed. It was therefore not possible to investigate more detailed differences in drinking patterns such as frequency of heavy episodic or “binge” drinking, or prevalence of alcohol use disorders. In addition, the maximum drink size option available in the questions on usual amount consumed per occasion was “three portions”. To this extent, it was not possible to discriminate heavier drinkers who usually drink more than three portions of alcohol per drinking occasion. There was a small percentage of participants in each site who reported drinking 3 portions of either beer, wine or spirits which suggests some degree of under-estimation of total volume of ethanol consumed is likely. The type of alcoholic beverage consumed under liquor and spirits included home-brewed alcohol with varying alcohol strengths which could potentially have been underestimated. However given the general limitations of using self-reported volume of ethanol from surveys as measures of actual ethanol consumption, where estimates are consistently lower compared with sales data[40–42], our measures seem appropriate for our purpose of examining broad differences in amount consumed between the study sites. Given the high levels of abstaining from alcohol particularly within Ghana we investigated associations with drinking any alcohol which does not take into account differences in drinking pattern among drinkers and therefore further work would be needed to understand the associations with drinking patterns in these populations.

Despite these limitations, our study provides original data on the drinking of Ghanaians in different locations and factors that may be contributing to these patterns. These findings suggest the need for policy makers to consider the different patterns when addressing the issue of alcohol consumption through public health campaigns and advocacy. Different strategies might be required in alcohol reduction campaigns among Ghanaians in different locations. A potentially effective strategy might include further exploration of the role of religious organisations in alcohol-related public health campaigns and how to reach those not attending religious services.

While we have shown migration is associated with differences in drinking behaviour, further work is needed to understand what factors drive these changes and conversely the role of cultural and religious factors in promoting lower alcohol consumption in some migrant groups compared with their host populations.

## Supporting information

S1 Table Characteristics of participants in London and Amsterdam with missing data on alcohol consumption. (DOCX)

S2 Table Characteristics of participants by study site (male). (DOCX)

S3 Table Characteristics of participants by study site (female). (DOCX)

S4 Table Association between drinking alcohol with socio-demographic and behavioural factors and measures of acculturation in men and women mutually adjusted for all variables incuding site for European countries. (DOCX)

## References

[1] Forouzanfar MH, Alexander L, Anderson HR, Bachman VF, Biryukov S, Brauer M, et al Global, regional, and national comparative risk assessment of 79 behavioural, environmental and occupational, and metabolic risks or clusters of risks in 188 countries, 1990–2013: a systematic analysis for the Global Burden of Disease Study 2013. Lancet. 2015;386(10010):2287–323. Epub 2015/09/15. 10.1016/S0140-6736(15)00128-2 ; PubMed Central PMCID: PMCPMC4685753. 26364544 PMC4685753

[2] Rehm J, Shield KD. Global alcohol-attributable deaths from cancer, liver cirrhosis, and injury in 2010. Alcohol research: current reviews. 2013;35(2):174–83. Epub 2013/01/01. ; PubMed Central PMCID: PMCPmc3908708. 24881325 10.35946/arcr.v35.2.07 PMC3908708

[3] World Health Organization. Global Status Report on Alcohol and Health Geneva:World Health Organization Available at: http://apps.who.int/iris/bitstream/10665/112736/1/9789240692763_eng.pdf. 2014.

[4] Rehm J, Room R, Graham K, Monteiro M, Gmel G, Sempos CT. The relationship of average volume of alcohol consumption and patterns of drinking to burden of disease: An overview. Addiction. 2003;98(9):1209–28. . 12930209 10.1046/j.1360-0443.2003.00467.x

[5] Rehm J, Taylor B, Patra J. Volume of alcohol consumption, patterns of drinking and burden of disease in the European region 2002. Addiction (Abingdon, England). 2006;101(8):1086–95. Epub 2006/07/28. 10.1111/j.1360-0443.2006.01491.x . 16869838

[6] Rehm J, Kailasapillai S, Larsen E, Rehm MX, Samokhvalov AV, Shield KD, et al A systematic review of the epidemiology of unrecorded alcohol consumption and the chemical composition of unrecorded alcohol. Addiction (Abingdon, England). 2014;109(6):880–93. Epub 2014/01/29. 10.1111/add.12498 . 24467748

[7] Kanteres F, Lachenmeier DW, Rehm J. Alcohol in Mayan Guatemala: consumption, distribution, production and composition of cuxa. Addiction (Abingdon, England). 2009;104(5):752–9. Epub 2009/02/14. 10.1111/j.1360-0443.2009.02507.x . 19215596

[8] Rehm J, Mathers C, Popova S, Thavorncharoensap M, Teerawattananon Y, Patra J. Global burden of disease and injury and economic cost attributable to alcohol use and alcohol-use disorders. Lancet. 2009;373(9682):2223–33. Epub 2009/06/30. 10.1016/S0140-6736(09)60746-7 . 19560604

[9] World Health Organization. WHO Expert Committee on Problems Related to Alcohol Consumption. Second report, Geneva. 2007. 17970166

[10] Babor T, Caetano R, Casswell S, Edwards G, Giesbrecht N, Graham K, et al Alcohol: No ordinary commodity—Research and Public Policy Oxford, UK: Oxford University Press; 2010.

[11] Shield KD, Rylett M, Gmel G, Gmel G, Kehoe-Chan TA, Rehm J. Global alcohol exposure estimates by country, territory and region for 2005—a contribution to the Comparative Risk Assessment for the 2010 Global Burden of Disease Study. Addiction (Abingdon, England). 2013;108(5):912–22. Epub 2013/01/26. 10.1111/add.12112 . 23347092

[12] Oyebode O, Pape UJ, Laverty AA, Lee JT, Bhan N, Millett C. Rural, urban and migrant differences in non-communicable disease risk-factors in middle income countries: a cross-sectional study of WHO-SAGE data. PLoS One. 2015;10(4):e0122747 Epub 2015/04/08. 10.1371/journal.pone.0122747 ; PubMed Central PMCID: PMCPMC4388413. 25849356 PMC4388413

[13] Chen HH, Chien LY. Ethnic Drinking Culture, Acculturation, and Enculturation in Relation to Alcohol Drinking Behavior Among Marriage-Based Male Immigrants in Taiwan. American journal of men's health. 2018:1557988318772744. Epub 2018/05/03. 10.1177/1557988318772744 . 29717913 PMC6142147

[14] Dumbili E. Changing Patterns of Alcohol Consumption in Nigeria: An Exploration of Responsible factors and Consequences. Medical Sociology online Available at: http://wwwmedicalsociologyonlineorg/MSoVol7Iss1/MSoVol7Iss1Art2/71_Art2html. 2013;7.

[15] Organisation WH. Global status report on alcohol and health 2014 Available at:http://wwwwhoint/substance_abuse/publications/global_alcohol_report/en/. 2014.

[16] Oshodin OG. Nigeria In Heath B. D. (Ed.), International handbook on alcohol and culture (First ed., pp. 213–223). Westport: Greenwood Press 1995.

[17] Luginaah I, Dakubo C. Consumption and impacts of local brewed alcohol (akpeteshie) in the Upper West Region of Ghana: a public health tragedy. Soc Sci Med. 2003;57(9):1747–60. Epub 2003/09/02. . 12948582 10.1016/s0277-9536(03)00014-5

[18] Luginaah I. Local gin (akpeteshie) and HIV/AIDS in the Upper West Region of Ghana: the need for preventive health policy. Health Place. 2008;14(4):806–16. Epub 2008/02/20. 10.1016/j.healthplace.2007.12.007 . 18282781

[19] Zemore SE. Acculturation and alcohol among Latino adults in the United States: a comprehensive review. Alcoholism, clinical and experimental research. 2007;31(12):1968–90. Epub 2007/11/24. 10.1111/j.1530-0277.2007.00532.x . 18034692

[20] Acculturation J.W. B. and adaptation in a new society. International Migration. 1992;30(1):69–85.

[21] McCullough Cosgrove J, LeCroy CW, Fordney M, Voelkel D. Considering the Role of Acculturation in Parent-Child Communication About Sexual Health. Am J Public Health. 2018;108(S1):S13–s4. Epub 2018/02/15. 10.2105/AJPH.2017.304222 ; PubMed Central PMCID: PMCPMC5813781. 29443559 PMC5813781

[22] Hosper K, Nierkens V, Nicolaou M, Stronks K. Behavioural risk factors in two generations of non-Western migrants: do trends converge towards the host population? Eur J Epidemiol. 2007;22(3):163–72. Epub 2007/03/06. 10.1007/s10654-007-9104-7 ; PubMed Central PMCID: PMCPMC2781098. 17334819 PMC2781098

[23] Lara M, Gamboa C, Kahramanian MI, Morales LS, Bautista DE. Acculturation and Latino health in the United States: a review of the literature and its sociopolitical context. Annu Rev Public Health. 2005;26:367–97. Epub 2005/03/12. 10.1146/annurev.publhealth.26.021304.144615 . 15760294 PMC5920562

[24] Agyemang C, Beune E, Meeks K, Owusu-Dabo E, Agyei-Baffour P, Aikins A, et al Rationale and cross-sectional study design of the Research on Obesity and type 2 Diabetes among African Migrants: the RODAM study. BMJ Open. 2014;4(3):e004877 Epub 2014/03/25. 10.1136/bmjopen-2014-004877 . 24657884 PMC3963103

[25] Galbete C, Nicolaou M, Meeks KA, de-Graft Aikins A, Addo J, Amoah SK, et al Food consumption, nutrient intake, and dietary patterns in Ghanaian migrants in Europe and their compatriots in Ghana. Food & nutrition research. 2017;61(1):1341809 Epub 2017/07/28. 10.1080/16546628.2017.1341809 ; PubMed Central PMCID: PMCPMC5510194. 28747862 PMC5510194

[26] Rosengren A, Hawken S, Ounpuu S, Sliwa PK, Zubaid M, Almahmeed WA, et al Association of psychosocial risk factors with risk of acute myocardial infarction in 11 119 cases and 13 648 controls from 52 countries (the INTERHEART study): Case-control study. Lancet. 2004;364(9438):953–62. 10.1016/S0140-6736(04)17019-0 15364186

[27] Berry JW. Immigration, acculturation, and adaptation. Applied Psychology: An International Review. 1997;46(1):5–34.

[28] Stevens GW VW, Pels TV, Crijnen AA,. Patterns of Psychological Acculturation in Adult Aolescent Moroccan Immigrants Living in the Netherlands. Journal of Cross-Cultural Psychology. 2004;35 (6):689–704.

[29] Erol A, Karpyak VM. Sex and gender-related differences in alcohol use and its consequences: Contemporary knowledge and future research considerations. Drug and alcohol dependence. 2015;156:1–13. Epub 2015/09/16. 10.1016/j.drugalcdep.2015.08.023 . 26371405

[30] Wilsnack RW, Vogeltanz ND, Wilsnack SC, Harris TR, Ahlstrom S, Bondy S, et al Gender differences in alcohol consumption and adverse drinking consequences: cross-cultural patterns. Addiction (Abingdon, England). 2000;95(2):251–65. Epub 2000/03/21. . 10723854 10.1046/j.1360-0443.2000.95225112.x

[31] Makela P, Gmel G, Grittner U, Kuendig H, Kuntsche S, Bloomfield K, et al Drinking patterns and their gender differences in Europe. Alcohol and alcoholism (Oxford, Oxfordshire) Supplement. 2006;41(1):i8–18. Epub 2006/10/13. 10.1093/alcalc/agl071 . 17030504

[32] Zemore SE. Acculturation and alcohol among Latino adults in the United States: A comprehensive review. Alcohol Clin Exp Res. 2007;31(12):1968–90. 10.1111/j.1530-0277.2007.00532.x . 18034692

[33] Martinez P, Roislien J, Naidoo N, Clausen T. Alcohol abstinence and drinking among African women: data from the World Health Surveys. BMC Public Health. 2011;11:160 Epub 2011/03/12. 10.1186/1471-2458-11-160 ; PubMed Central PMCID: PMCPMC3061917. 21392398 PMC3061917

[34] Ferreira-Borges C, Rehm J, Dias S, Babor T, Parry CD. The impact of alcohol consumption on African people in 2012: an analysis of burden of disease. Trop Med Int Health. 2016;21(1):52–60. Epub 2015/10/09. 10.1111/tmi.12618 . 26448195

[35] World Health Organisation. European Status Report on Alcohol and Health 2014. Geneva: 2014.

[36] Bernards S, Graham K, Kuendig H, Hettige S, Obot I. 'I have no interest in drinking': a cross-national comparison of reasons why men and women abstain from alcohol use. Addiction (Abingdon, England). 2009;104(10):1658–68. Epub 2009/08/18. 10.1111/j.1360-0443.2009.02667.x ; PubMed Central PMCID: PMCPMC2891671. 19681798 PMC2891671

[37] Lucchetti G, Koenig HG, Pinsky I, Laranjeira R, Vallada H. Religious beliefs and alcohol control policies: a Brazilian nationwide study. Revista brasileira de psiquiatria (Sao Paulo, Brazil: 1999). 2014;36(1):4–10. Epub 2013/12/19. 10.1590/1516-4446-2012-1051 . 24346358

[38] Bloomfield K, Grittner U, Kramer S, Gmel G. Social inequalities in alcohol consumption and alcohol-related problems in the study countries of the EU concerted action 'Gender, Culture and Alcohol Problems: a Multi-national Study'. Alcohol and alcoholism (Oxford, Oxfordshire) Supplement. 2006;41(1):i26–36. Epub 2006/10/13. 10.1093/alcalc/agl073 . 17030500

[39] Grittner U, Kuntsche S, Graham K, Bloomfield K. Social inequalities and gender differences in the experience of alcohol-related problems. Alcohol and alcoholism (Oxford, Oxfordshire). 2012;47(5):597–605. Epub 2012/05/01. 10.1093/alcalc/ags040 ; PubMed Central PMCID: PMCPMC3417684. 22542707 PMC3417684

[40] World Health Organisation. International Guide for Monitoring Alcohol Consumption and Related Harm Geneva, Switzerland: Department of Mental Health and Substance Dependence, 2000.

[41] Casswell S, Zhang JF, Wyllie A. The importance of amount and location of drinking for the experience of alcohol-related problems. Addiction. 1993;88(11):1527–34. . 8286998 10.1111/j.1360-0443.1993.tb03138.x

[42] Russell M, Welte JW, Barnes GM. Quantity-frequency measures of alcohol consumption: beverage-specific vs global questions. Br J Addiction. 1991;86(4):409–17. . 10.1111/j.1360-0443.1991.tb03418.x 2054535

