# Supplementary material for: Differences in alcohol consumption and drinking patterns in Ghanaians in Europe and Africa: The RODAM Study
Source: PLoS One. 2018 Nov 2;13(11):e0206286. doi: 10.1371/journal.pone.0206286 (PMC6214514; doi:10.1371/journal.pone.0206286)
Supplement: S1 Table — (DOCX) [file pone.0206286.s001.docx]

**S1 Table. Characteristics of participants in London and Amsterdam with missing data on alcohol consumption**

|  | | Amsterdam | | | | | | London | | | |
| --- | --- | --- | --- | --- | --- | --- | --- | --- | --- | --- | --- |
|  |  | Data on alcohol consumption | | | No data on alcohol consumption | | | Data on alcohol consumption | | No data on alcohol consumption | |
|  |  | N | (%) | | N | (%) | | N | (%) | N | (%) |
|  |  |  |  | |  |  | |  |  |  |  |
| Sex | Male | 359 | (39.5) | | 187 | (38.7) | | 170 | (40.0) | 181 | (36.9) |
|  | Female | 551 | (60.6) | | 296 | (61.3) | | 255 | (60.0) | 309 | (63.1) |
|  | Chi square (df) p value | 0.07 (1)p=0.79 | | | | | | 0.90 (1) p=0.34 | | | |
| Age | 25-34 | 63 | (6.9) | | 51 | (10.6) | | 41 | (9.7) | 62 | (12.7) |
|  | 35-44 | 262 | (28.8) | | 108 | (22.4) | | 96 | (22.6) | 116 | (23.7) |
|  | 45-54 | 366 | (40.2) | | 195 | (40.4) | | 140 | (32.9) | 190 | (38.8) |
|  | 55-70 | 219 | (24.1) | | 129 | (26.7) | | 148 | (34.8) | 122 | (24.9) |
|  | Test for trend | 0.13(10 p=0.72 | | | | | | 6.9 (1) p=0.009 | | | |
| Education | never been to school/elementary school only | 313 | (34.7) | | 169 | (36.0) | | 39 | (9.4) | 43 | (9.1) |
|  | lower vocational school/secondary | 536 | (59.5) | | 272 | (58.0) | | 243 | (58.3) | 271 | (57.3) |
|  | higher level/university | 52 | (5.8) | | 28 | (6.0) | | 135 | (32.4) | 159 | (33.6) |
|  | Chi square(df) p value | 0.28 (2) p=0.87 | | | | | | 0.16 (2)p=0.93 | | | |
| Marital status | married | 192 | (21.5) | | 79 | (16.6) | | 277 | (68.2) | 324 | (70.7) |
|  | cohabiting | 207 | (23.1) | | 84 | (17.7) | | 8 | (2.0) | 10 | (2.2) |
|  | never married | 240 | (26.8) | | 150 | (31.6) | | 47 | (11.6) | 42 | (9.2) |
|  | divorced/separated | 243 | (27.2) | | 161 | (33.9) | | 61 | (15.0) | 63 | (13.8) |
|  | widowed | 13 | (1.5) | | 1 | (0.2) | | 13 | (3.2) | 19 | (4.2) |
|  | Chi square(df) p value | 19.9 (4) p=0.001 | | | | | | 2.2 (4) p=0.70 | | | |
| Frequency of attending religious services | Once a week | 589 | (70.0) | | 279 | (62.0) | | 354 | (89.2) | 387 | (89.0) |
|  | At least once a month but not every week | 95 | (11.3) | | 60 | (13.3) | | 23 | (5.8) | 22 | (5.1) |
|  | Less than once a month | 32 | (3.8) | | 24 | (5.3) | | 9 | (2.3) | 1 | (0.2) |
|  | Never/no current religion | 125 | (14.9) | | 87 | (19.3) | | 11 | (2.8) | 25 | (5.8) |
|  | Chi square(df) p value | 9.0 (3) p=0.03 | | | | | | 11.6 (3) p=0.009 | | | |
| Smoking | Current smoker | 38 | (4.2) | | 25 | (5.2) | | 2 | (0.5) | 2 | (0.4) |
|  | Never smoker | 796 | (88.0) | | 403 | (84.1) | | 400 | (94.8) | 461 | (95.1) |
|  | Ex-smoker | 71 | (7.9) | | 51 | (10.7) | | 20 | (4.7) | 22 | (4.5) |
|  | Chi square(df) p value | 4.0 (2) p=0.13 | | | | | | 0.04 (2) p=0.98 | | | |
| Psycho-social stress | Never experience stress | 436 | (48.8) | | 225 | (47.5) | | 200 | (49.6) | 239 | (54.3) |
|  | Some periods of stress and home or work | 323 | (36.1) | | 169 | (35.7) | | 160 | (39.7) | 156 | (35.5) |
|  | Several periods or stress at home or work/permanent stress at home or work | 135 | (15.1) | | 80 | (16.9) | | 43 | (10.7) | 45 | (10.2) |
|  | Chi square(df) p value | 0.75 (2) p=0.69 | | | | | | 1.9 (2) p=0.38 | | | |
| Years since migration | 1-5 | 83 | (9.4) | | 24 | (5.1) | | 44 | (11.1) | 55 | (12.1) |
|  | 5-9 | 120 | (13.6) | | 28 | (5.9) | | 63 | (15.8) | 81 | (17.9) |
|  | 10+ | 682 | (77.1) | | 419 | (89.0) | | 291 | (73.1) | 317 | (70.0) |
|  | Chi square(df) p value | 28.8 (2) p<0.001 | | | | | | 1.03 (2) p=0.60 | | | |
| Acculturation (ethnic identity) | More acculturated | 657 | (72.2) | | 332 | (68.7) | | 217 | (51.1) | 275 | (56.1) |
|  | Less acculturated | 253 | (27.8) | | 151 | (31.3) | | 208 | (48.9) | 215 | (43.9) |
|  | Chi square(df) p value | 1.84 (1)p=0.18 | | | |  | | 2.35 (1) p=0.13 | | | |
| Acculturation (cultural orientation) | More acculturated | 669 | (73.5) | 366 | | | (75.8) | 352 | (82.8) | 421 | (85.9) |
|  | Less acculturated | 241 | (26.5) | | 117 | (24.2) | | 73 | (17.2) | 69 | (14.1) |
|  | Chi square(df) p value | 0.84 (1) p=0.36 | | | | | | 1.66 (1) p=0.20 | | | |
| Acculturation (social networks) | More acculturated | 611 | (67.1) | | 328 | (67.9) | | 344 | (80.9) | 401 | (81.8) |
|  | Less acculturated | 299 | (32.9) | | 155 | (32.1) | | 81 | (19.1) | 89 | (18.2) |
|  | Chi square (df) p value | 0.08 (1) p=0.77 | | | | | | 0.12 (1)p=0.73 | | | |

*Missing data on covariates excluded
