# Supplementary material for: Differences in alcohol consumption and drinking patterns in Ghanaians in Europe and Africa: The RODAM Study
Source: PLoS One. 2018 Nov 2;13(11):e0206286. doi: 10.1371/journal.pone.0206286 (PMC6214514; doi:10.1371/journal.pone.0206286)
Supplement: S2 Table — (DOCX) [file pone.0206286.s002.docx]

**Supplementary Table 2 (S2) Characteristics of participants by study site (male)**

|  | |  | | | | | **Europe (all sites)** | | | | | | | | | | | | | | | **Amsterdam** | | | | | | | | | | | **Berlin** | | **London** | | | | | | | | | | | | **Urban Ghana** | | | | | | | | | | | | | | **Rural Ghana** | | | | | | | | | | | | | | |
| --- | --- | --- | --- | --- | --- | --- | --- | --- | --- | --- | --- | --- | --- | --- | --- | --- | --- | --- | --- | --- | --- | --- | --- | --- | --- | --- | --- | --- | --- | --- | --- | --- | --- | --- | --- | --- | --- | --- | --- | --- | --- | --- | --- | --- | --- | --- | --- | --- | --- | --- | --- | --- | --- | --- | --- | --- | --- | --- | --- | --- | --- | --- | --- | --- | --- | --- | --- | --- | --- | --- | --- | --- | --- | --- | --- |
|  | |  | | | | | N=819 | | | | | | | | | | | |  | | | | N=359 | | | | | | | N=290 | | | | | | | | | | | N=170 | | | | | | | | | N=408 | | | | | | | | | | | | | | | N=392 | | | | | | | | | | |
|  |  |  | | | | | N | | | | | | (%) | | | | | |  | | | | N | | | (%) | | | | N | | | (%) | | | | | N | | | | | | | (%) | | | | | N | | | | | | | | (%) | | | | | | | N | | | | | | | | | | (%) |
| Age | | 25-34 | | | | | | 98 | | | | | (12.0) | | | | | |  | | | | 19 | | | (5.3) | | | | 55 | | | (19.0) | | | | 24 | | | | | | | | (14.1) | | | | | 77 | | | | | | | | (18.9) | | | | | | | 82 | | | | | | | | | | (20.9) |
|  |  | 35-44 | | | | | | 180 | | | | | (22.0) | | | | | |  | | | | 78 | | | (21.7) | | | | 64 | | | (22.1) | | | | | 38 | | | | | | | (22.4) | | | | | 88 | | | | | | | | (21.6) | | | | | | | 95 | | | | | | | | | | (24.2) |
|  |  | 45-54 | | | | | | 285 | | | | | (34.8) | | | | | |  | | | | 141 | | | (39.3) | | | | 95 | | | (32.8) | | | | | 49 | | | | | | | (28.8) | | | | | 114 | | | | | | | | (27.9) | | | | | | | 94 | | | | | | | | | | (24.0) |
|  |  | 55-70 | | | | | | 256 | | | | | (31.3) | | | | | |  | | | | 121 | | | (33.7) | | | | 76 | | | (26.2) | | | | | 59 | | | | | | | (34.7) | | | | | 129 | | | | | | | | (31.6) | | | | | | | 121 | | | | | | | | | | (30.9) |
| Education | | Missing | | | | | | | 6 | | | | (0.7) | | | | | |  | | | | 2 | | | (0.6) | | | | 1 | | | (0.3) | | | | | | | | 3 | | | | (1.8) | | | | | 21 | | | | | | | | | (5.2) | | | | | | 23 | | | | | | | (5.9) | | | |
|  |  | never been to school/elementary school only | | | | | | 104 | | | | | (12.7) | | | | | |  | | | | 76 | | | (21.2) | | | | 18 | | | (6.2) | | | | | | | | 10 | | | | (5.9) | | | | | 89 | | | | | | | | | (21.8) | | | | | | 149 | | | | | | | (38.0) | | | |
|  |  | lower vocational school/secondary | | | | | | 553 | | | | | (67.5) | | | | | |  | | | | 249 | | | (69.4) | | | | 221 | | | (76.2) | | | | | | | | 83 | | | | (48.8) | | | | | 260 | | | | | | | | | (63.7) | | | | | | 197 | | | | | | | (50.3) | | | |
|  |  | higher level/university | | | | | 156 | | | | | | (19.1) | | | | | |  | | | | 32 | | | (8.9) | | | | 50 | | | (17.2) | | | | | | | | 74 | | | | (43.5) | | | | | 38 | | | | | | | | | (9.3) | | | | | | 23 | | | | | | | (5.9) | | | |
| Marital status | | Missing | | | | | 14 | | | | | | (1.7) | | | | | |  | | | | 6 | | | (1.7) | | | | 2 | | | (0.7) | | | | | | | | 6 | | | | (3.5) | | | | | 21 | | | | | | | | (5.2) | | | | | | | 24 | | | | | | | (6.1) | | | |
|  |  | married | | | | | 368 | | | | | | (44.9) | | | | | |  | | | | 100 | | | (27.9) | | | | 140 | | | (48.3) | | | | | | | | 128 | | | | (75.3) | | | | | 281 | | | | | | | | (68.9) | | | | | | | 239 | | | | | | | | | (61.0) | |
|  |  | cohabiting | | | | | 134 | | | | | | (16.4) | | | | | |  | | | | 100 | | | (27.9) | | | | 31 | | | (10.7) | | | | | | | | 3 | | | | (1.8) | | | | | 24 | | | | | | | | (5.9) | | | | | | | 66 | | | | | | | (16.8) | | | |
|  |  | never married | | | | | 173 | | | | | | (21.1) | | | | | |  | | | | 82 | | | (22.8) | | | | 74 | | | (25.5) | | | | | | | | 17 | | | | (10.0) | | | | | 58 | | | | | | | | (14.2) | | | | | | | 27 | | | | | | | (6.9) | | | |
|  |  | divorced/separated | | | | | 124 | | | | | | (15.1) | | | | | |  | | | | 68 | | | (18.9) | | | | 43 | | | (14.8) | | | | | | | | 13 | | | | (7.7) | | | | | 21 | | | | | | | | (5.2) | | | | | | | 32 | | | | | | | (8.2) | | | |
|  |  | widowed | | | | | 6 | | | | | | (0.7) | | | | | |  | | | | 3 | | | (0.8) | | | | 0 | | | (0.7) | | | | | | | | 3 | | | | (1.8) | | | | | 3 | | | | | | | | (0.7) | | | | | | | 4 | | | | | | | (1.0) | | | |
| Frequency of attending religious service | | Missing | | | | | 33 | | | | | | (4.0) | | | | | |  | | | | 20 | | | (5.6) | | | | 0 | | | (0.0) | | | | | | | | 13 | | | | (7.7) | | | | | 20 | | | | | | | | (4.9) | | | | | | | 25 | | | | | | | (6.4) | | | |
|  |  | Once a week | | | | | 465 | | | | | | (56.8) | | | | | |  | | | | 204 | | | (56.8) | | | | 128 | | | (44.1) | | | | | | | | 133 | | | | (78.2) | | | | | 257 | | | | | | | | (63.0) | | | | | | | 220 | | | | | | | (56.1) | | | |
|  |  | At least once a month but not every week | | | | | 102 | | | | | | (12.5) | | | | | |  | | | | 48 | | | (13.4) | | | | 44 | | (15.2) | | | | | | | | | 10 | | | | (5.9) | | | | | 9 | | | | | | (2.2) | | | | | | | | | | | | 17 | | | | | (4.3) | | |
|  |  | Less than once a month | | | | | 49 | | | | | | (6.0) | | | | | |  | | | | 20 | | | (5.6) | | | | 21 | | | (7.2) | | | | | | | | 8 | | | (4.7) | | | | | | 6 | | | | | | | (1.5) | | | | | | | | | | | 6 | | | | | | (1.5) | |
|  |  | Never /No current religion | | | | | 170 | | | | | | (20.8) | | | | | |  | | | | 67 | | | (18.7) | | | | 97 | | | (33.5) | | | | | | | | 6 | | | (3.5) | | | | | | 116 | | | | | | | (28.4) | | | | | | | | | | 124 | | | | | | | (31.6) | |
| Smoking | | Missing |  | | | | 2 | | | | | | (0.2) | | | | | | 1 | | | | | | | | (0.3) | | | 0 | | (0.0) | | | | | | | 1 | | | | | (0.6) | | | | | | 20 | | | | | | | (4.9) | | | | | | | | | 23 | | | | | | | | (5.9) | |
|  |  | Current smoker |  | | | | 76 | | | | | | (9.3) | | | | | | 30 | | | | | | | | (8.4) | | | 44 | | (15.2) | | | | | | | 2 | | | | | (1.2) | | | | | | 13 | | | | | | | (3.2) | | | | | | | | | 22 | | | | | | | | (5.6) | |
|  |  | Never smoker |  | | | | 641 | | | | | | (78.3) | | | | | | 284 | | | | | | | | (79.1) | | | 203 | | (70.0) | | | | | | | 154 | | | | | (90.6) | | | | | | 315 | | | | | | | (77.2) | | | | | | | | | 292 | | | | | | | | (74.5) | |
|  |  | Ex-smoker |  | | | | 100 | | | | | | (12.2) | | | | | | 44 | | | | | | | | (12.3) | | | 43 | | (14.8) | | | | | | | 13 | | | | | (7.7) | | | | | | 60 | | | | | | | (14.7) | | | | | | | | | 55 | | | | | | | | (14.0) | |
| Psycho-social stress | | Missing |  | | | | 12 | | | | | | (1.5) | | | | | | 3 | | | | | | | | (0.8) | | | 0 | | (0.0) | | | | | | | 9 | | | | | (5.3) | | | | | | 21 | | | | | | | (5.2) | | | | | | | | | 30 | | | | | | | | (7.7) | |
|  |  | Never experience stress |  | | | | 391 | | | | | | (47.7) | | | | | | 178 | | | | | | | | (49.6) | | | 132 | | (45.5) | | | | | | | 81 | | | | | (47.7) | | | | | | 144 | | | | | | | (35.3) | | | | | | | | | 92 | | | | | | | | (23.5) | |
|  |  | Some periods of stress and home or work |  | | | | 311 | | | | | | (38.0) | | | | | | 128 | | | | | | | | (35.7) | | | 116 | | (40.0) | | | | | | | 67 | | | | | (39.4) | | | | | | 182 | | | | | | | (44.6) | | | | | | | | | 223 | | | | | | | | (56.9) | |
|  |  | Several periods or stress at home or work/permanent stress at home or work |  | | | | 105 | | | | | | (12.8) | | | | | | 50 | | | | | | | | (13.9) | | | 42 | | (14.5) | | | | | | | 13 | | | | | (7.7) | | | | | | 61 | | | | | | | (15.0) | | | | | | | | | 47 | | | | | | | | (12.0) | |
| Years since migration | | Missing | | | | | 26 | | | | | | (3.2) | | | | | |  | | | | 8 | | | | (2.2) | | | 5 | | | (1.7) | | | | | | | | 13 | | | | (7.7) | | | | |  | | | | | | | |  | | | | | | |  | | | | | |  | | | | |
|  |  | 1-5 | | | | | 133 | | | | | | (16.2) | | | | | |  | | | | 36 | | | | (10.0) | | | 76 | | | (26.2) | | | | | | | | 21 | | | | (12.4) | | | | |  | | | | | | | |  | | | | | | |  | | | | | |  | | | | |
|  |  | 5-9 | | | | | 71 | | | | | | (8.7) | | | | | |  | | | | 34 | | | | (9.5) | | | 13 | | | (4.5) | | | | | | | | 24 | | | | (14.1) | | | | |  | | | | | | | |  | | | | | | |  | | | | | |  | | | | |
|  |  | 10+ | | | | | 589 | | | | | | (71.9) | | | | | |  | | | | 281 | | | | (78.3) | | | 196 | | | (67.6) | | | | | | | | 112 | | | | (65.9) | | | | |  | | | | | | | |  | | | | | | |  | | | | | |  | | | | |
| Acculturation (ethnic identity) | | More Integrated | | | | | 501 | | | | | | | (61.2) | | | | |  | | | | 258 | | | | (71.9) | | | 145 | | | (50.0) | | | | | | | 98 | | | (57.7) | | | | | | |  | |  | | | | | | | |  | | | | | | | | | |  | | | | | |
|  | | Less Integrated | | | | 318 | | | | | | (38.8) | | | | |  | | | | 101 | | | | | (28.3) | | | 145 | | | (50.0) | | | | | | | 72 | | | |  | | | | (42.4) | |  | | | | |  | | | | | | | | |  | | | | | | | | | | | | |
| Acculturation (cultural orientation) | | More Integrated | | 623 | | | | | | (76.1) | | | | | |  | | | | 269 | | | | (74.9) | | | | | | 207 | (71.4) | | | | | | | 147 | | | | (86.5) | | | | | | | | | | |  | | | | | | | | |  | | | | | | | | | | | | | |
|  |  | Less Integrated | | 196 | | | | | | (23.9) | | | | | |  | | | | 90 | | | | (25.1) | | | | | | 83 | (28.6) | | | | | | | 23 | | | | (13.5) | | | | | |  | | | | |  | | | | | | | | |  | | | | | | | | | | | | | |
| Acculturation (social networks) | More Integrated | | | | 596 | | | | | | (72.8) | | | |  | | | 375 | | | | | | | (68.1) | | | 185 | | | | | | (77.1) | | 206 | | | | | | | | | | (80.8) | | | | |  | | | |  | | | | | | | | |  | | | | |  | | | | | | |
|  | Less Integrated | | | | 223 | | | | | | (27.2) | | | |  | | | 176 | | | | | | | (31.9) | | | 55 | | | | | | (22.9) | | 49 | | | | | | | | | | (19.2) | | | | |  | | | |  | | | | | | | | |  | | | | |  | | | | | | |
