# Supplementary material for: Differences in alcohol consumption and drinking patterns in Ghanaians in Europe and Africa: The RODAM Study
Source: PLoS One. 2018 Nov 2;13(11):e0206286. doi: 10.1371/journal.pone.0206286 (PMC6214514; doi:10.1371/journal.pone.0206286)
Supplement: S3 Table — (DOCX) [file pone.0206286.s003.docx]

**S3 Table. Characteristics of participants by study site (female)**

|  |  | **Europe**  **(all sites)** | | | | **Amsterdam** | | | **Berlin** | | **London** | | | | | | **Urban Ghana** | | | | | | | **Rural Ghana** | | |
| --- | --- | --- | --- | --- | --- | --- | --- | --- | --- | --- | --- | --- | --- | --- | --- | --- | --- | --- | --- | --- | --- | --- | --- | --- | --- | --- |
|  | | N=1046 | | | | N=551 | | | N=240 | | N=255 | | | | | | N=1013 | | | | | | | N=602 | | |
|  |  | N | | | (%) | N | (%) | | N | (%) | N | | | | (%) | | N | | | (%) | | | | N | | (%) |
| Age | 25-34 | 94 | | | (9.0) | 44 | (8.0) | | 33 | (13.8) | 17 | | | | (6.7) | | 191 | | | (18.9) | | | | 120 | | (19.9) |
|  | 35-44 | 316 | | | (30.2) | 184 | (33.4) | | 74 | (30.8) | 58 | | | | (22.8) | | 279 | | | | | (27.5) | | 141 | | (23.4) |
|  | 45-54 | 405 | | | (38.7) | 225 | (40.8) | | 89 | (37.1) | 91 | | | | (35.7) | | 298 | | | | | (29.4) | | 173 | | (28.7) |
|  | 55-70 | 231 | | | (22.1) | 98 | (17.8) | | 44 | (18.3) | 89 | | | | (34.9) | | 245 | | | | | (24.2) | | 168 | | (27.9) |
| Education | Missing | 13 | | | (1.2) | 7 | (1.3) | | 1 | (0.4) | 5 | | | | (2.0) | | 19 | | | (1.9) | | | | 42 | | (7.0) |
|  | never been to school/elementary school only | 295 | | | (28.2) | 237 | (43.0) | | 29 | (12.1) | 29 | | | | (11.4) | | 513 | | | (50.6) | | | | 366 | | (60.8) |
|  | lower vocational school/secondary | 639 | | | (61.1) | 287 | (52.1) | | 192 | (80.0) | 160 | | | | (62.8) | | 453 | | | (44.7) | | | | 182 | | (30.2) |
|  | higher level/university | 99 | | | (9.5) | 20 | (3.6) | | 18 | (7.5) | 61 | | | | (23.9) | | 28 | | | (2.8) | | | | 12 | | (2.0) |
| Marital status | Missing | 23 | | | (2.2) | 9 | (1.6) | | 1 | (0.4) | 13 | | | | (5.1) | | 25 | | | (2.5) | | | | 42 | | (7.0) |
|  | married | 356 | | | (34.0) | 92 | (16.7) | | 115 | (47.9) | 149 | | | | (58.4) | | 523 | | | (51.6) | | | | 290 | | (48.2) |
|  | cohabiting | 126 | | | (12.1) | 107 | (19.4) | | 14 | (5.8) | 5 | | | | (2.0) | | 89 | | | (8.8) | | | | 90 | | (15.0) |
|  | never married | 225 | | | (21.5) | 158 | (28.7) | | 37 | (15.4) | 30 | | | | (11.8) | | 76 | | | (7.5) | | | | 21 | | (3.5) |
|  | divorced/separated | 290 | | | (27.7) | 175 | (31.8) | | 67 | (27.9) | 48 | | | | (18.8) | | 169 | | | (16.7) | | | | 84 | | (14.0) |
|  | widowed | 26 | | | (2.5) | 10 | (1.8) | | 6 | (2.5) | 10 | | | | (3.9) | | 131 | | | (12.9) | | | | 75 | | (12.5) |
| Frequency of attending religious services | Missing | 66 | | | (6.3) | 49 | (8.9) | | 2 | (0.8) | 15 | | | | (5.9) | | 19 | | | (1.9) | | | | 43 | | (7.1) |
|  | Once a week | 777 | | | (74.3) | 385 | (69.9) | | 171 | (71.3) | 221 | | | | (86.7) | | 720 | | | (71.1) | | | | 362 | | (60.1) |
|  | At least once a month but not every week | 92 | | | (8.8) | 47 | (8.5) | | 32 | (13.3) | 13 | | | | (5.1) | | 25 | | | (2.5) | | | | 26 | | (4.3) |
|  | Less than once a month | 22 | | | (2.1) | 12 | (2.2) | | 9 | (3.8) | 1 | | | | (0.4) | | 7 | | | (0.7) | | | | 9 | | (1.5) |
|  | Never/no current religion | 89 | | | (8.5) | 58 | (10.5) | | 26 | (10.8) | 5 | | | | (2.0) | | 242 | | | (23.9) | | | | 162 | | (26.9) |
| Smoking | Missing | 6 | | | (0.6) | 4 | (0.7) | | 0 | (0.0) | 2 | | | | (0.8) | | 20 | | | | (2.0) | | | 43 | | (7.1) |
|  | Current smoker | 15 | | | (1.4) | 8 | (1.5) | | 7 | (2.9) | 0 | | | | (0.0) | | 1 | | | | (0.1) | | | 0 | | (0.0) |
|  | Never smoker | 976 | | | (93.3) | 512 | (92.9) | | 218 | (90.8) | 246 | | | | (96.5) | | 972 | | | | (96.0) | | | 554 | | (92.0) |
|  | Ex-smoker | 49 | | | (4.7) | 27 | (4.9) | | 15 | (6.3) | 7 | | | | (2.8) | | 20 | | | | (2.0) | | | 5 | | (0.8) |
| Psycho-social stress | Missing | 26 | | | (2.5) | 13 | (2.4) | | 0 | (0.0) | 13 | | | | (5.1) | | 26 | | | (2.6) | | | | 45 | | (7.5) |
|  | Never experience stress | 486 | | | (46.5) | 258 | (46.8) | | 109 | (45.4) | 119 | | | | (46.7) | | 312 | | | (30.8) | | | | 127 | | (21.1) |
|  | Some periods of stress and home or work | 367 | | | (35.1) | 195 | (35.4) | | 79 | (32.9) | 93 | | | | (36.5) | | 542 | | | (53.5) | | | | 310 | | (51.5) |
|  | Several periods or stress at home or work/permanent stress at home or work | 167 | | | (16.0) | 85 | (15.4) | | 52 | (21.7) | 30 | | | | (11.8) | | 133 | | | (13.1) | | | | 120 | | (19.9) |
| Years since migration | Missing | 36 | | | (3.4) | 17 | (3.1) | | 5 | (2.1) | 14 | | | | | (5.5) | |  | |  | | | |  | |  |
|  | 1-5 | 112 | | | (10.7) | 47 | (8.5) | | 42 | (17.5) | 23 | | | | | (9.0) | |  | |  | | | |  | |  |
|  | 5-9 | 149 | | | (14.2) | 86 | (15.6) | 24 | | (10.0) | | | 39 | | | (15.3) | |  | |  | | |  | | | |
|  | 10+ | 749 | (71.6) | | | 401 | (72.8) | 169 | | (70.4) | | | | 179 | | (70.2) | | |  | |  | | |  |  | |
| Acculturation (ethnic identity) | More integrated | 623 | (59.6) | | | 399 | (72.4) | 105 | | (43.8) | | 119 | | | | (46.7) | |  | | | | | | | | |
|  | Less integrated | 423 | (40.4) | | | 152 | (27.6) | | 135 | (56.3) | 136 | | | | | (53.3) | |  | | | | | | | | |
| Acculturation (cultural orientation) | More integrated | 765 | (73.1) | | | 400 | (72.6) | | 160 | (66.7) | 205 | | | | (80.4) | |  | | | | | | | | | |
|  | Less integrated | 281 | (26.9) | | | 151 | (27.4) | | 80 | (33.3) | 50 | | | | (19.6) | |  | | | | | | | | | |
| Acculturation (social networks) | More integrated | 766 | | (73.2) | | 375 | (68.1) | | 185 | (77.1) | 206 | | | | (80.8) | |  | | | | | | | | | |
|  | Less integrated | 280 | | (26.8) | | 176 | (31.9) | | 55 | (22.9) | 49 | | | | (19.2) | |  | | | | | | | | | |
