# Supplementary material for: Differences in alcohol consumption and drinking patterns in Ghanaians in Europe and Africa: The RODAM Study
Source: PLoS One. 2018 Nov 2;13(11):e0206286. doi: 10.1371/journal.pone.0206286 (PMC6214514; doi:10.1371/journal.pone.0206286)
Supplement: S4 Table — (DOCX) [file pone.0206286.s004.docx]

**S4 Table. Association between drinking alcohol with socio-demographic and behavioural factors and measures of acculturation in men and women mutually adjusted for all variables**

|  | | Europe | | | | | | Urban Ghana | | | | | | Rural Ghana | | | |
| --- | --- | --- | --- | --- | --- | --- | --- | --- | --- | --- | --- | --- | --- | --- | --- | --- | --- |
|  |  | Male (n=739) | | | | Female (n=892) | | Male (n=384) | | Female(n=978) | | | | Male (n=359) | | Female (n=551) | |
|  |  | OR | | (95 % CI) | | OR | (95% CI) | OR | (95% CI) | OR | | | (95% CI) | OR | (95% CI) | OR | (95% CI) |
| Age | 25-34 | 0.85 | | (0.44, 1.65) | | 0.85 | (0.49,1.47) | 0.99 | (0.47,2.10) | 0.53 | | | (0.32, 0.85) | 1.27 | (0.64, 2.54) | 1.10 | (0.63, 1.91) |
|  | 35-44 | 1.00 | | (ref) | | 1.00 | (ref) | 1.00 | (ref) | 1.00 | | | (ref) | 1.00 | (ref) | 1.00 | (ref) |
|  | 45-54 | 1.02 | | (0.64, 1.63) | | 1.55 | (1.10, 2.19) | 0.62 | (0.34, 1.15) | 0.92 | | | (0.63,1.34) | 1.10 | (0.57, 2.13) | 0.64 | (0.39, 1.06) |
|  | 55-70 | 1.37 | | (0.82, 2.30) | | 1.74 | (1.15, 2.64) | 0.71 | (0.39, 1.30) | 0.68 | | | (0.44, 1.05) | 1.20 | (0.64, 2.24) | 0.51 | (0.30, 0.88) |
|  | Test for linear trend | P=0.15 | |  | | P=0.002 |  | P=0.26 |  | P=0.73 | | |  | P=0.97 |  | P=0.003 |  |
| Education | Never been to school/elementary school | 1.21 | | (0.71,2.04) | | 1.10 | (0.79,1.53) | 1.82 | (1.07, 3.12) | 1.59 | | | (1.17, 2.16) | 1.20 | (0.73, 1.95) | 1.03 | (0.68,1.56) |
|  | Lower vocational school/secondary | 1.00 | | (ref) | | 1.00 | (ref) | 1.00 | (ref) | 1.00 | | | (ref) | 1.00 | (ref) | 1.00 | (ref) |
|  | Higher level/university | 1.46 | | (0.75, 2.83) | | 1.05 | (0.59,1.86) | 1.99 | (0.87, 4.56) | 2.10 | | | (0.89, 4.99) | 0.70 | (0.28, 1.80) | 0.49 | (0.10, 2.33) |
|  | Test for linear trend | P=0.26 | |  | | P=0.71 |  | P=0.04 |  | P=0.002 | | |  | P=0.97 |  | P=0.77 |  |
| Marital status | Married | 1.00 | | (ref) | | 1.00 | (ref) | 1.00 | (ref) | 1.00 | | | (ref) | 1.00 | (ref) | 1.00 | (ref) |
|  | Cohabiting | 1.56 | | (0.91, 2.67) | | 1.25 | (0.76, 2.07) | 1.63 | (0.65, 4.04) | 1.77 | | | (1.07, 2.94) | 1.30 | (0.70, 2.40) | 1.49 | (0.89, 2.51) |
|  | Never Married | 1.32 | | (0.81, 2.16) | | 1.26 | (0.84, 1.88) | 1.00 | (0.46, 2.13) | 1.41 | | | (0.75, 2.66) | 1.10 | (0.44, 2.74) | 0.12 | (0.02. 0.65) |
|  | Divorced/Separated | 0.75 | | (0.45, 1.26) | | 1.21 | (0.84, 1.74) | 1.12 | (0.44, 2.84) | 1.35 | | | (0.91, 2.01) | 3.42 | (1.31, 8.93) | 1.25 | (0.72, 2.16) |
|  | Widowed | Perfect | | prediction | | 1.24 | (0.52, 2.97) | 0.68 | (0.06, 8.14) | 1.28 | | | (0.79, 2.09) | 2.47 | (0.24, 25.25) | 1.98 | (1.10, 3.55) |
|  | Test for heterogeneity | P=0.10 | |  | | P=0.82 |  | P=0.87 |  | P=0.17 | | |  | P=0.09 |  | P=0.002 |  |
| Frequency of attending religious service | Once a week | 1.00 | | (ref) | | 1.00 | (ref) | 1.00 | (ref) | 1.00 | | | (ref) | 1.00 | (ref) | 1.00 | (ref) |
|  | At least once a month but not every week | 4.49 | | (2.48, 8.13) | | 1.25 | (0.78, 2.01) | 1.34 | (0.34, 5.38) | 2.13 | | | (0.91, 5.00) | 2.58 | (0.84, 7.90) | 1.88 | (0.77, 4.62) |
|  | Less than once a month | 4.59 | | (2.03, 10.37) | | 2.37 | (0.83, 6.78) | 0.48 | (0.08, 3.00) | 0.93 | | | (0.18, 4.97) | 1.89 | (0.31, 11.51) | 0.54 | (0.10, 2.83) |
|  | Never /No current religion | 3.91 | | (2.37,6.45) | | 1.74 | (1.05, 2.89) | 1.54 | (0.95, 2.49) | 1.41 | (1.01, 1.97) | | | 2.20 | (1.33, 3.64) | 0.82 | (0.54, 1.26) |
|  | Test for linear trend | P<0.001 | |  | | P=0.01 | | P=0.09 |  | P=0.04 | | | | P=0.002 |  | P=0.36 | |
| Smoking status | Never smoker | 1.00 | | (ref) | | 1.00 | (ref) | 1.00 | (ref) | 1.00 | | (ref) | | 1.00 | (ref) | 1.00 | (ref) |
|  | Current smoker | 5.89 | | (2.24, 15.54) | | Perfect | Prediction | 2.27 | (0.71, 7.27) | Perfect | | Prediction | | 4.84 | (1.33, 17.60) | - |  |
|  | Ex-smoker | 1.27 | | (0.76, 2.15) | | 1.62 | (0.82, 3.21) | 2.16 | (1.19, 3.93) | 2.52 | | (1.01, 6.32) | | 2.23 | (1.13, 4.40) | 9.14 | (0.83, 100.59) |
|  | Test for heterogeneity | P=0.0001 | |  | | - |  | P=0.21 |  | - | |  | | P=0.003 |  | P=0.04 |  |
| Psych- social stress | Never experience stress | 1.00 | | (ref) | | 1.00 | (ref) | 1.00 | (ref) | 1.00 | | (ref) | | 1.00 | (ref) | 1.00 | (ref) |
|  | Some periods of stress and home or work | 1.31 | | (0.91, 1.89) | | 1.15 | (0.85, 1.56) | 0.77 | (0.48, 1.24) | 1.05 | | (0.75, 1.47) | | 1.22 | (0.73, 2.06) | 0.87 | (0.54, 1.40) |
|  | Several periods of stress at home or work/ Permanent stress at home or work | 1.13 | | (0.66, 1.92) | | 1.04 | (0.70,1.53) | 1.35 | (0.72, 2.53) | 1.27 | | (0.79, 2.05) | | 1.78 | (0.82, 3..85) | 0.79 | (0.44, 1.41) |
|  | Test for trend | P=0.33 | |  | | P=0.65 |  | P=0.65 |  | P=0.37 | |  | | P=0.15 |  | P=0.42 |  |
| Years since Migration | 0-4 years | 0.71 | | (0.42,1.20) | | 0.69 | (0.43, 1.09) |  |  |  | |  | |  |  |  |  |
|  | 5-9 years | 0.53 | | (0.29, 0.99) | | 0.77 | (0.52, 1.16) |  |  |  | |  | |  |  |  |  |
|  | 10 or more years | 1.00 | | (ref) | | 1.00 | (ref) |  |  |  | |  | |  |  |  |  |
|  | Test for linear trend | P=0.10 | |  | | P=0.07 |  |  |  |  | |  | |  |  |  |  |
| Acculturation (ethnic identity) | More acculturated | 1.00 (ref) | |  | | 1.00 | (ref) |  |  |  | |  | |  |  |  |  |
|  | Less acculturated | 0.82 | | (0.57, 1.18) | | 0.97 | (0.72,1.31) |  |  |  | |  | |  |  |  |  |
| Acculturation (cultural orientation) | More acculturated | 1.00 (ref) | |  | | 1.00 | (ref) |  |  |  | |  | |  |  |  |  |
|  | Less acculturated | 1.16 | | (0.76,1.76) | | 0.87 | (0.63, 1.20) |  |  |  | |  | |  |  |  |  |
| Acculturation (social networks) | More acculturated | 1.00 (ref) | | |  | 1.00 | (ref) |  |  |  | |  | |  |  |  |  |
|  | Less acculturated | 1.03 | (0.70, 1.52) | | | 1.22 | (0.89, 1.67) |  |  |  | |  | |  |  |  |  |

*Model 1: Adjusted for age + education + marital status + smoking status + frequency of attending religious services + psycho-social stress (+site+ years of migration + acculturation for European countries)
